# AtZIK4, a MAPK kinase kinase, positively regulates phosphate uptake gene expression to modulate phosphate starvation tolerance in *Arabidopsis*

**DOI:** 10.1515/biol-2025-1361

**Published:** 2026-08-27

**Authors:** Hongying Deng, Qian Yang, Shirong Zhao, Xuyang Lei, Liye Zhu

**Affiliations:** Hebei Center for Industrial Energy-Saving and Pollution Control Research, Hebei Vocational University of Technology and Engineering, Xingtai, Hebei, 054000, China; School of Medicine, Nankai University, Tianjin, 300071, China

**Keywords:** phosphate starvation stress, MAPK kinase, *Arabidopsis thaliana*, anthocyanin

## Abstract

The mitogen-activated protein kinase (MAPK) cascade is a highly conserved signal transduction module in eukaryotes. We investigated the function of ZIK4, a MAPK kinase, in the phosphate starvation response of Arabidopsis. Phenotypic analysis revealed that the *zik4* mutant had stronger tolerance to phosphate starvation than that to the wild type. However, the kinase domain was still expressed in *zik4*. The CRISPR/Cas9 system was used to construct two independent knockout mutants *zik4-c3* and *zik4-c15* by targeting two different sites in the AtZIK4 kinase domain. Physiological measurements showed that the phosphate content in *zik4-c3* and *zik4-c15* was lower than that in the wild type. Expression pattern analysis revealed that ZIK4 was induced to express under phosphate starvation. Quantitative real-time PCR results showed that the expression levels of multiple key phosphate uptake-related genes were significantly up-regulated in the *zik4* mutant. These results indicate that ZIK4, as a phosphate starvation-induced MAPKKK, promotes phosphate accumulation in Arabidopsis seedlings by upregulating the expression levels of phosphate uptake-related genes. These findings not only elucidate the regulatory role of AtZIK4 kinase during abiotic stress and expand the low-phosphate (LP) signaling cascade in *Arabidopsis thaliana*, but also providing promising genetic targets to enhance crop performance under phosphorus-limited conditions.

## 1 Introductions

As an important component of macromolecules, phosphorus (P) is a fundamental part of nucleic acids, coenzymes, phospholipids, and sugar phosphates [1], 2]. It plays a crucial role in numerous essential biological functions, particularly in energy metabolism. In addition, it plays a crucial role in signal transduction cascades, promoting communication between cells and enabling them to respond to external stimuli. In addition, it also participates in the regulation of enzymes, which are key catalysts for biochemical reactions [3]. Plants selectively uptake phosphorus (P) as inorganic phosphate (Pi), which has low mobility in soil and is often difficult to plants [4]. Therefore, the concentration of available Pi significantly affects plant growth and productivity in various soil types. To maintain a growing global population, a large amount of phosphorus (P) fertilizer is needed. It comes from non-renewable resources and is expected to decline significantly in the next century. However, the utilization efficiency of phosphorus supplied in fertilizers is quite low. Globally, due to significant losses and inefficiencies in the process from extraction to consumption, only one-fifth of the phosphorus is ultimately consumed [5]. To address this, a deeper understanding of how plants perceive and respond to changes in phosphate (Pi) concentrations at the molecular level can promote crop development, increase phosphorus acquisition and utilization under limited availability while maintaining yield [2].

Plants developed under reduced availability phosphate (Pi) exhibit a series of responses to Pi scarcity, including changes in developmental, physiological, and biochemical processes. The accumulation of anthocyanin in the aboveground parts of plants, manifested a purple, is a typical response to decreased Pi levels and various other stress factors [6]. Anthocyanins are a vibrant natural pigment with colors ranging from red to purple [7]. They have multiple functions in plant growth and adaptation, including attracting pollinators and seed dispersers, protecting plants from UV radiation, affecting auxin transport, and neutralizing free radicals under stressful conditions [8].

Chip analysis was conducted on the transcriptome of *Arabidopsis thaliana* subjected to phosphate (Pi) deprivation. Genes responsive to Pi starvation stress were identified based on their expression levels. The functional classification of these genes includes a variety of metabolic pathways, ion transport mechanisms, signal transduction processes, transcriptional regulatory activities, and other aspects related to growth and development [9]. These genes include transcription factors, non-coding RNAs, SPX subfamily proteins, and proteins involved in protein SUMOylation, phosphorylation, and dephosphorylation [9], [10], [11].

The external introduction of phosphate analogues such as phosphite can alleviate the physiological, biochemical, and molecular response mechanisms of plants experiencing Pi deficiency stress. This indicates that Pi is a direct signal regulating the Pi starvation response [12]. To perceive and transmit this Pi signal, plants utilize specific messenger molecules. Inositol pyrophosphate InsP8 functions as a signaling molecule that transmits information on cellular phosphorus levels to the SPX domain to regulate cellular Pi homeostasis. It is therefore crucial for plants to perceive cellular phosphorus status [13], 14]. The process of plant perception and transmission of phosphorus signals is still under investigation.

The mitogen-activated protein kinase (MAPK) cascade system is a highly conserved signaling pathway in eukaryotes. It is the main mechanism of signal transduction in these organisms. The basic signaling system of the MAPK cascade includes three tiers of kinases: MAPKKK, MAPKK, and MAPK. Sequential phosphorylation promotes the specific amplification of external stimulus signals conveyed to the cell [15]. MKK9s-MPK3/MPK6 has been shown to regulate the Pi response in plants [16], 17]. However, how MKK9-MPK3/MPK6 is activated in Pi starvation stress and whether MAPKKK participates in the low-phosphate response is still unknown.

Arabidopsis genome encodes 80 MAPKKKs, including 21 MEKKs (MAPK/ERK Kinase Kinases), 11 ZIKs, and 48 RAFs (Rapidly Accelerated Fibrosarcoma kinases) [18]. However, most of them are uncharacterized. The ZIK subfamily, also named as WNK (With-No-lysine (K) kinase), lacks the conserved catalytic lysine in the subdomain II of other MAPK kinase kinases [19]. Analysis of ZIKs/WNKs in plants is only conducted in a few cases. The limited functional information indicates that ZIKs/WNKs are involved in regulating the circadian clock. This will affect flowering time, responding to ionic and osmotic stresses, and modulating glucose signaling [19], [20], [21], [22].

Based on this, the working hypothesis is that ZIK4 acts as an upstream MAPKKK, activating the MKK9-MPK3/MPK6 cascade to mediate Pi starvation signaling. The aim is to elucidate the functional role of ZIK4 in response to phosphate (Pi) deficiency during planting seedling stage. This study emphasizes that ZIK4 in Arabidopsis plays an important role in the response of plants to phosphate (Pi) deficiency during the seedling stage. The expression of this gene is induced by Pi starvation stress, which promotes the adaptive response of plants by modulating the expression of genes related to Pi starvation responses.

## 2 Materials and methods

### 2.1 Plant materials and growth conditions

The wild-type plants in this study were the Columbia-0 ecotype. T-DNA insertion mutant of *AtZIK4* (salk_015778) and other *mapkkk* mutants with the Col-0 background were provided by Professor Dongtao Ren from China Agricultural University.

Arabidopsis (*A. thaliana*) seeds were surface sterilized with 75 % (v/v) ethanol for 1 min, then sterilize with 2.5 % (v/v) sodium hypochlorite containing 0.1 % Triton X-100 for 10 min, and rinsed five times with sterile distilled water. To break seed dormancy and ensure synchronous germination, the sterilized seeds were cold-treated at 4 °C in the dark for 2 days. Then, the seeds were sown on 90 mm × 20 mm Glass Petri dishes 1/2 MS medium supplemented with 625 μM Pi (supplied as KH_2_PO_4_), 1 % (w/v) sucrose, and 1 % (w/v) agar (Sigma-Aldrich, Cat# A7926). Each treatment uses three repeating plates, and the entire experiment was independently repeated three times. Plants were cultivated in a growth room at 22 °C under a 16 h light/8 h dark photoperiod with a light intensity of 100 μmol m^−2^ s^−1^ for 7 d.

For phosphate (Pi) treatment experiments, 7-day-old seedlings of uniform size were carefully transferred to MS medium (1.25 mM Pi) or low-phosphate (LP) medium (50 μM Pi). The LP medium was prepared by modifying the composition of MS medium [23], with the following modifications: the final concentration of KH_2_PO_4_ was reduced to 50 μM, and agar was replaced with 1 % (w/v) agarose (Promega, Cat# V3121) to minimize Pi contamination by the gelling agent. After transfer, the seedlings were grown on the respective media in the additional days of each experiment. Photographs were taken at designated time points. After seedlings harvesting, they were immediately frozen in liquid nitrogen and stored at −80 °C to subsequent measurements of anthocyanin and Pi content measurements.

For soil growth, seedings were transferred from 1/2 MS medium to soil under 16/8 h light and dark cycle at 22 °C in green house. Soil-grown plants were used for genetic crossing and seed harvesting.

### 2.2 Quantification of total Pi

To determine Pi content, the analysis was conducted following a previously established protocol [24]. Seven-day-old seedlings grown on 1/2 MS medium were transferred to MS (1.25 mM Pi) or LP (50 μM Pi) medium for another 7 d. Each biological replicate consists of 30–40 seedlings treated with MS treatment or 50–60 seedlings treated with LP, collected from a single Petri dish, with three biological replicates per treatment. After treatment, shoots and roots were separated. Then they were washed three times with distilled water, absorbed dry, and dried in a forced-air drying oven at 80 °C for at least 8 h. Dry weights were measured using an analytical balance (Sartorius BSA224S).

Dried samples were placed into pre-cleaned porcelain crucibles (30 mL; cleaned with 0.1 M HCl overnight, rinsed with distilled and Milli-Q water) and ashed in a muffle furnace (Nabertherm L9/11/SKM) at 310 °C for 1 h, then calcined at 575 °C for 5 h. The ash was dissolved in 1 mL of 0.1 M HCl at room temperature for 1 h, then transferred to 1.5 mL microcentrifuge tubes and left overnight to ensure complete dissolution.

For Pi quantification, samples were centrifuged at 12,000×*g* for 5 min. Aliquots of supernatant (200 μL for MS-treated shoots, 400 μL for LP-treated shoots, and 500 μL for roots) were brought to 900 μL with 0.1 M HCl. Then it was mixed with 100 μL of ammonium vanadomolybdate reagent and incubated at room temperature for 15 min. Absorbance at 410 nm was measured using a microplate reader (BioTek Epoch 2). Serial dilutions of 1 mM KH_2_PO_4_ were prepared and processed with the samples to generate the standard curve.

Pi content is calculated as follows:

(1) $$\text{Pi content }\left(\mu \text{mol}/g\text{ DW}\right)=\left(c\times 1000\right)/\left(V\times \text{DW}\right)$$

where c is the Pi concentration (mM) from the standard curve; 1000 is the conversion factor from mM to μM per mL; V is the aliquot volume (μL) taken for measurement, and DW is the dry weight (g) of the tissue sample. Each biological replicate was measured three times.

### 2.3 Anthocyanin measurement

To determine anthocyanin, 7-day-old seedlings were transferred to MS (1.25 mM Pi) or LP (50 μM Pi) medium for another 7 d. Three biological replicates were prepared for each treatment. Each replicate consisted of 30 seedlings for LP treatment or 20 seedlings for MS treatment, and pooled from a single Petri dish. Shoots were harvested and weighed using an analytical balance (Sartorius BSA224S, Sartorius AG, Göttingen, Germany), and frozen immediately in liquid nitrogen. Anthocyanin was analyzed using a predefined protocol [25].

The frozen tissues were ground in liquid nitrogen and 1 mL of extraction buffer (1 % HCl in methanol, v/v) was added and incubated at 4 °C overnight in the dark with continuous shaking for extraction. The extracts were centrifuged at 12,000 rpm for 5 min using a refrigerated centrifuge (Eppendorf 5424R, Eppendorf AG, Hamburg, Germany). 200 μL of supernatant was transferred to a 96-well microplate, and absorbance was measured at 530 nm (anthocyanin maximum absorption) and 657 nm (chlorophyll correction) using a microplate reader (BioTek Epoch 2, BioTek Instruments, Winooski, VT, USA).

Anthocyanin content was calculated as follows:

(2) $$\text{Anthocyanin content}\left(\text{unit}/g\right)=\left(A530-0.25\times A657\right)/\text{FW}$$

where A_530_ is absorbance at 530 nm; A_657_ is absorbance at 657 nm (chlorophyll interference); 0.25 is the empirically determined correction coefficient for chlorophyll contribution at 530 nm, and FW is fresh weight (g). Each biological replicate was measured three times.

### 2.4 Construction of CRISPR/Cas9 targeting vector

To generate knockout mutants of *AtZIK4*, a dual-target CRISPR/Cas9 system was used. The binary vector pHSE401 carrying the *Cas9* gene controlled by the parsley ubiquitin promoter was provided by Professor Qijun Chen (China Agricultural University). Two independent single-guide RNA (sgRNA) target sites were designed in the kinase domain of AtZIK4 using the online CRISPR-P tool (http://crispr.hzau.edu.cn/CRISPR2/). The dual sgRNA expression cassette was assembled via Golden Gate cloning. A four-primer PCR strategy was adopted to amplify the sgRNA cassette using the pCBC-DT1T2 vector (diluted 100-fold) as the template. The PCR product was purified and digested with the restriction enzyme *Bsa*I, and ligated into the pHSE401 vector. The ligation product was transformed into *E. coli* DH5α competent cells, and positive clones were selected on kanamycin-containing (50 μg/mL) LB plates. Colony PCR was carried out with vector-specific primers, followed by Sanger sequencing to confirm correct insertion. Then the verified construct was introduced into Agrobacterium tumefaciens strain GV3101 for subsequent Arabidopsis transformation. The primer sequences used for sgRNA cassette construction are shown in Table 1.

**Table 1:** Primers used for CRISPR/Cas9-mediated knockout system.

Primer name	Primer sequence (5′–3′)
*WNK1DT1*-BsF	ATA​TAT​GGT​CTC​GAT​TGG​TGG​ATC​ACA​GGA​GG
*WNK1DT1*-F_0_	TGG​TGG​ATC​ACA​GGA​GGA​TCA​GTT​TTA​GAG​CT
*WNK1DT2*-R_0_	AAC​ATG​CTG​CTC​ACT​GCG​TCG​GCA​ATC​TCT​TA
*WNK1DT2*-BsR *WNK1DT1*-BsF-1 *WNK1DT1*-F_0_–1 *WNK1DT2*-R_0_-1 *WNK1DT2*-BsR-1	ATTATTGGTCTCGAAACATGCTGCTCACTGCGTCGGC ATATATGGTCTCGATTGGTTAACGGGAATCAAGGAGGTT TGGTTAACGGGAATCAAGGAGGTTTTAGAGCT AACGAGTTCATGGCACCTGAAGCAATCTCTTA ATTATTGGTCTCGAAACGAGTTCATGGCACCT

### 2.5 SDS-PAGE and western blot analysis

Protein extraction, SDS-PAGE, and Western blot analysis were performed according to established protocols [26], 27] with modifications. Total protein was extracted from approximately 100 mg frozen tissues using ice-cold extraction buffer (50 mM Tris-HCl pH 7.5, 150 mM NaCl, 1 mM EDTA, 1 % Triton X-100, 1 mM PMSF, and protease inhibitor cocktail) and centrifuged at 12,000×*g* for 15 min at 4 °C using a refrigerated centrifuge (Eppendorf 5424R, Eppendorf AG, Hamburg, Germany). Protein concentration was determined using the Bradford assay (Bio-Rad Laboratories, Hercules, CA, USA).

Equal amounts of protein (5–15 μg per lane) were separated on a 10 % separating gel with a 5 % stacking gel using the Mini-PROTEAN Tetra electrophoresis system (Bio-Rad Laboratories). Electrophoresis was carried out at 80 V for 30 min through the stacking gel, and then separated by 120 V for 60 min through the separating gel. Then, proteins were transferred onto a nitrocellulose membrane (Merck Millipore, Darmstadt, Germany) using the Bio Rad Semi Dry Transfer Cell at 13 V for 30 min.

After transfer, the membranes were blocked with 5 % non-fat milk in TBST at room temperature for 1 h incubated with the primary antibody at 4 °C overnight, and then with HRP-conjugated secondary antibody (diluted 1:10,000) at room temperature for 1 h. The signal was detected using an enhanced chemiluminescence (ECL) substrate (Thermo Scientific, Waltham, MA, USA) and imaged with KODAK medical X-ray processor 102.

### 2.6 RNA isolation and real-time quantitative RT-PCR (Q-PCR)

Total RNA was extracted from samples using Trizol reagent (Invitrogen). RNA concentration was measured using a micro-volume UV-visible spectrophotometer (Nano Drop ND 1000). 2.5 μg of total RNA was used for reverse transcription. The synthesis of the first-strand cDNA utilized M-MLV reverse transcriptase (Promega), with oligo dT (16) serving as the primer and total RNA functioning as the template. To assess Pi-responsive gene expression, real-time Q-PCR was carried out combined with SYBR Green Mix (Takara, Dalian, China). The amplification process conducted in real time using a 7500 real-time PCR system (Applied Biosystems). *Ubiquitin5* expression levels were used as the internal control. Gene expression levels were calculated using the 2^−ΔΔCT^ method [28]. The primer sequences are shown in Table 2. All primers were designed using Primer3 software (v.4.1.0) and validated for target specificity via NCBI Primer-BLAST against the *A. thaliana* genome. The lengths of the *ZIK4, PHT1;1, PHT1;4*, *PHT1;4*, *MYB62, PHO1, At4*, *IPS1, DFR, UBQ5* amplicon is 163 bp, 210 bp, 188 bp, 160 bp, 150 bp, 503 bp, 186 bp, 155 bp, 192 bp and 149 bp. The RefSeq accession numbers for the amplified transcripts are as follows: *ZIK4* NM_111363.4, *PHT1;1* NM_123701.4, *PHT1;4* NM_129452.4, *WRKY75* NM_121311.5, *MYB62* NM_105503.3, *PHO1* NM_113246.5, *At4* NM_180427.2, *IPS1* NM_180219.1, *DFR* NM_123645.4, *UBQ5* NM_116090.3. All primers used in this SYBR Green I-based qPCR study are standard, unmodified DNA oligonucleotides without any fluorescent labels or chemical modifications. All standard primers were purified by PAGE (Polyacrylamide Gel Electrophoresis) to ensure high specificity in qPCR.

**Table 2:** Primers for quantitative real-time PCR.

Primers	Primer sequences (5–3′)
*ZIK4*-F *ZIK4*-R *PHT1;1*-F *PHT1;1*-R	CGCACCATCTTCCTGACT GGATTATACGCCCATCCA GTAGACGCAGGATACCCACCAG CAAAACCAAACATCGCACTCC
*PHT1;4*-F	CTC​GCT​TAT​TGT​GTT​GGG​TGT​AG
*PHT1;4*-R	AAC​GAA​AAT​TAT​TAC​AAA​AGG​CG
*WRKY75*-F	GAT​CAG​AAG​GTT​GTT​CGA​AAA​GC
*WRKY75*-R	CTC​CTA​GGG​AAC​TTG​TTG​TTC​TTG​AC
*MYB62*-F	GTG​CTG​GGC​TAA​AGA​GAA​CTG​GG
*MYB62*-R	CAA​TCT​TGG​ACC​ACC​TAT​TAC​CCC
*PHO1*–F *PHO1*-R *At4*-F *At4*-R *IPS1*–F *IPS1*-R *DFR*-F *DFR*-R *UBQ5*-F *UBQ5*-R	TAAGGAGATGGTGGGACGAA TTAACCGTCTGAGTCCCTGTC GTGTGTGAATGGAGCGATGAAG CTAAACCGGAAACAAAGTAAACACG GACTGCGAGTTTTGTTTATCTCCC CAACCCAAACATAATGAAGAAAAACA CCCGGTAATTTGAAGAAAGTACAAC CGGCTTTATCACTTCGTTCTCAG CTCCTTCTTTCTGGTAAACGT GGTGCTAAGAAGAGGAAGAAT

### 2.7 Statistical analysis

All experiments were conducted using three independent biological replicates, and repeated three times. Data are presented as mean ± standard deviation (SD). Statistical significance between two groups was determined using Student’s *t*-test (two-tailed, unpaired) in GraphPad Prism 8.0. Differences were considered statistically significant at *P* < 0.05 (*), *P* < 0.01 (**).

## 3 Results

### 3.1 ZIK4 mutants are insensitive to Pi starvation stress

**Figure 1:**
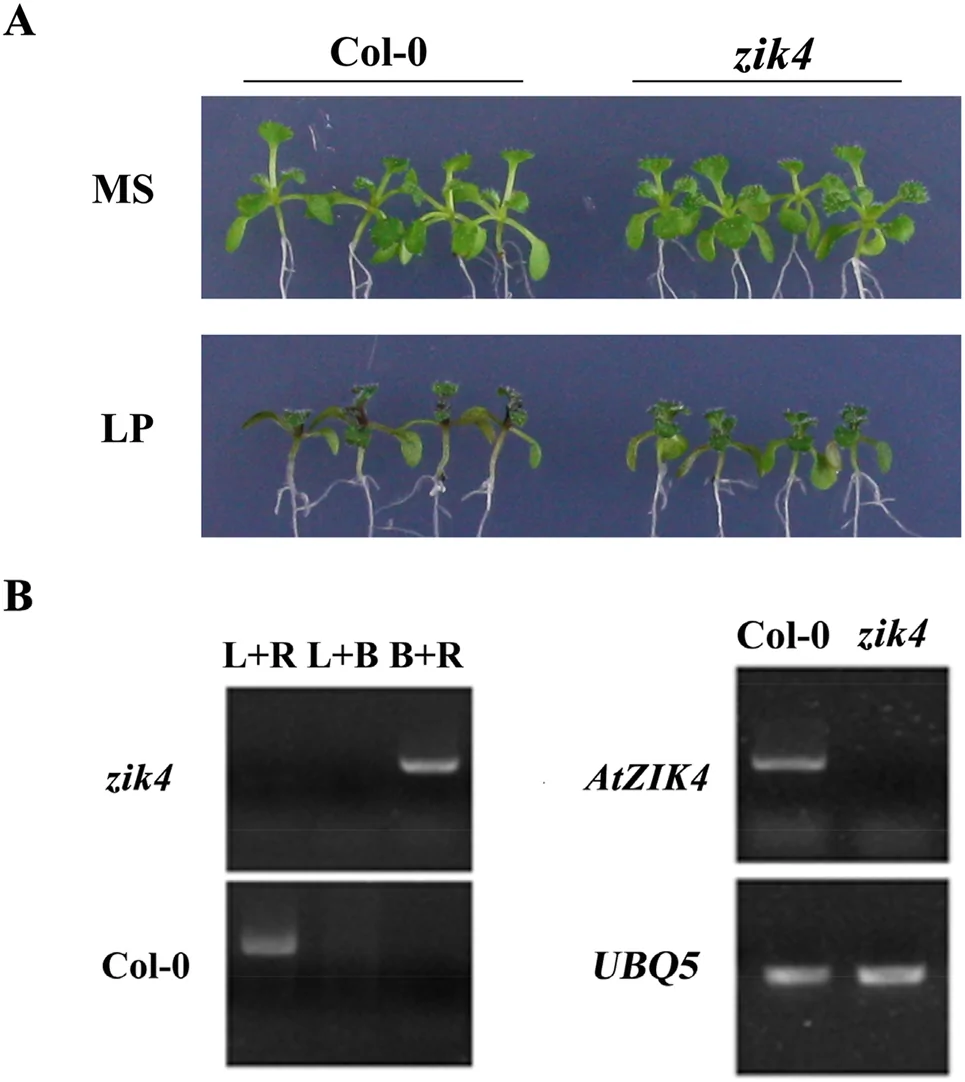
Identification of *zik4* mutant. (A) Phenotype of *zik4* mutant seedlings under phosphate starvation treatment. Col-0 and *zik4* seedlings vertically cultured on 1/2 MS medium for 7 d were transplanted to MS and LP media respectively, followed by another 7-day culture before photographing. Photographs were taken using a digital camera (Canon EOS 600D, Canon Inc., Tokyo, Japan). (B) Genomic PCR identification results indicated that *zik4* was a T-DNA insertion homozygous mutant. L, left border primer for T-DNA insertion site identification in mutant genotyping; R, right border primer for T-DNA insertion site identification in mutant genotyping; B, border primer (internal/central primer) for T-DNA insertion site identification in mutant genotyping. RT-PCR was performed to analyze the transcription level of *AtZIK4* in wild-type and mutant plants, with *UBQ5* as the internal reference.

To identify MAPKKK genes associated with Pi starvation stress response, a systematic screening of the MAPKKK T-DNA insertion mutants exposed to Pi starvation stress was conducted. Then they were compared with the wild type (Col-0) under the same growth. A mutant termed *zik4* was selected for further analysis, in which the expression of *ZIK4* (AT3G04910) was completely eliminated (Figure 1). The identified *zik4* mutant seeds and Col-0 seeds were evenly sown on 1/2MS medium. After 7 d of vertical light cultivation, the seedlings were transferred to Pi sufficient (MS) and Pi deficient (LP) medium for further cultivation for 7 d. Phenotypic observation showed that there was no significant difference between *zik4* mutant and Col-0 on MS medium. On LP medium, the aerial portion of Col-0 turned purple, and the *zik4* mutant did not have significant purple change (Figure 1A). In addition, there was a little difference in root architecture under LP treatment: wild-type Col-0 seedlings showed a decrease in primary root elongation and an increase in lateral root density; the *zik4* mutant showed slightly weaker inhibition of primary root growth and a slight reduction in lateral root formation compared with Col-0 under the same conditions.

**Figure 2:**
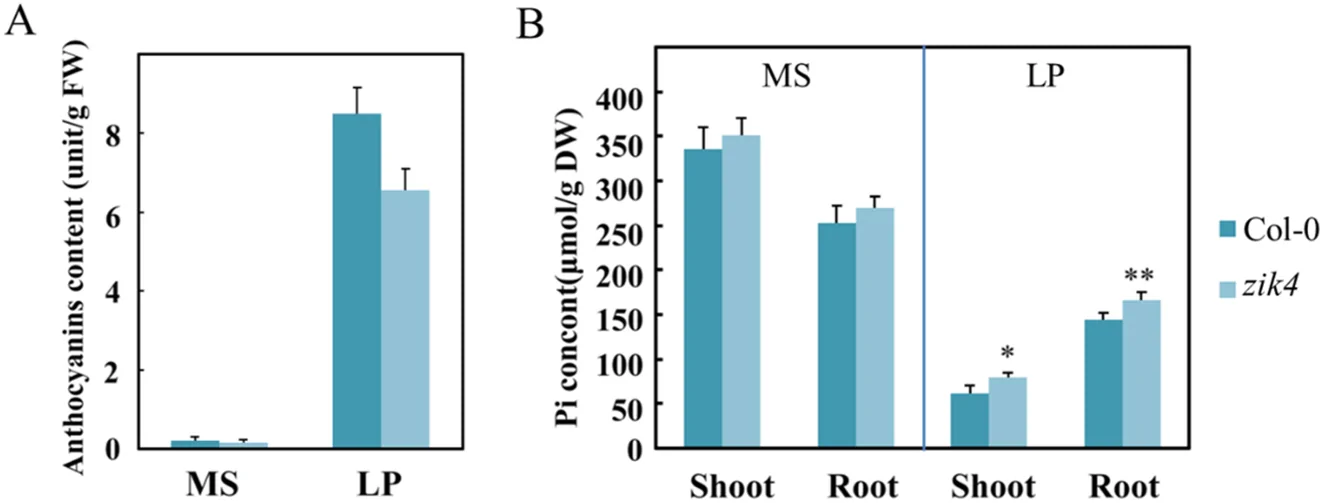
Enhanced tolerance to phosphate starvation stress of *zik4* mutant seedling. Col-0 and *zik4* seedlings vertically cultured on 1/2 MS medium for 7 d were transplanted to MS and LP media respectively for another 7 d of cultivation. Plant materials were harvested for phenotypic analysis respectively. The results are the mean values, with error bars indicating the standard deviation. All experiments were repeated with three times (statistical differences between the mutant and wild-type were determined by Student’s *t*-test, * indicates *P* < 0.05, ** indicates *P* < 0.01). (A) Determination results of anthocyanin content in Col-0 and *zik4* seedlings (B) determination results of Pi content in shoots and roots of Col-0 and *zik4* seedlings. MS, Murashige and skoog medium (Pi-sufficient, 1.25 mM Pi); LP, low-phosphate medium (Pi-deficient, 50 μM Pi).

The accumulation of anthocyanin and Pi content can reflect the adaptation of seedlings to phosphate starvation stress. The detection on anthocyanin contents detection revealed that Col-0 and *zik4* seedlings grown on MS medium contained lower but equivalent amounts of anthocyanin. When Col-0 and *zik4* seedlings were grown on LP medium, the anthocyanin content in their seedlings significantly increased. However, the anthocyanin level in *zik4* seedlings was 22.7 % lower than that in Col-0 seedlings (Figure 2(A)).

Seedlings were treated under the same conditions, and roots and shoots were harvested for Pi content determination. Although there was no significant difference in Pi content between the mutant and wild type on MS medium, the Pi content of the *zik4* mutant is slightly higher than that of the wild type. On LP medium, the difference between Col-0 and *zik4* was significant. Pi content of the mutant in shoots was 28.7 % higher than that of the wild type, and 15.4 % higher in the roots (Figure 2(B)). The above results indicate that the *zik4* mutant is more tolerant to phosphate starvation stress.

**Figure 3:**
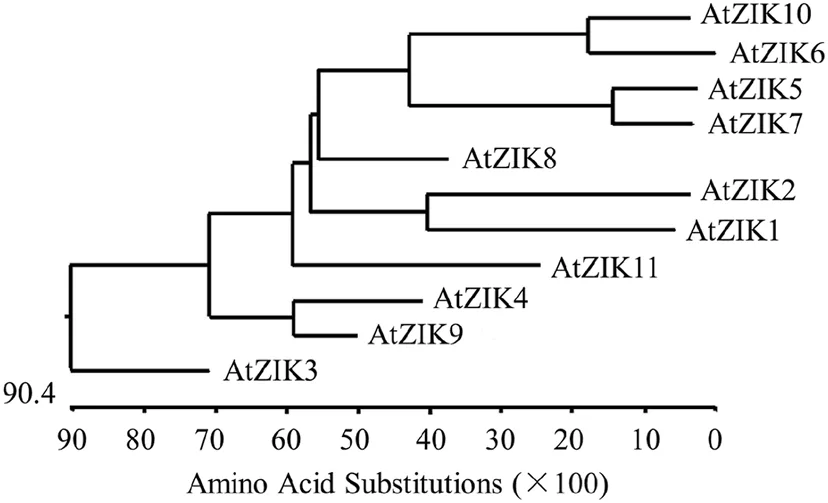
Phylogenetic analysis of the AtZIK family in *Arabidopsis thaliana*. The phylogenetic tree was constructed using MEGA7 software with the neighbor-joining algorithm to analyze the evolutionary relationships and sequence similarities in all members of the *Arabidopsis thaliana* AtZIK family. The scale bar indicates the genetic distance.

The Arabidopsis genome encodes a total of 11 ZIK genes, ranging from AtZIK1 to AtZIK11 [29]. Cluster analysis of the amino acid sequences of all ZIK family proteins (Figure 3) revealed that AtZIK4 shares the highest homology and closest phylogenetic relationship with AtZIK9. However, the T-DNA insertion mutant of *zik9* had no significant phenotypic difference from the wild type under LP treatment. This indicates that AtZIK4 is involved in the response of Arabidopsis to phosphate starvation stress, and AtZIK9 does not play a biological role in this process.

### 3.2 Isolation and phenotypic observation of AtZIK4 CRISPR mutants

**Figure 4:**
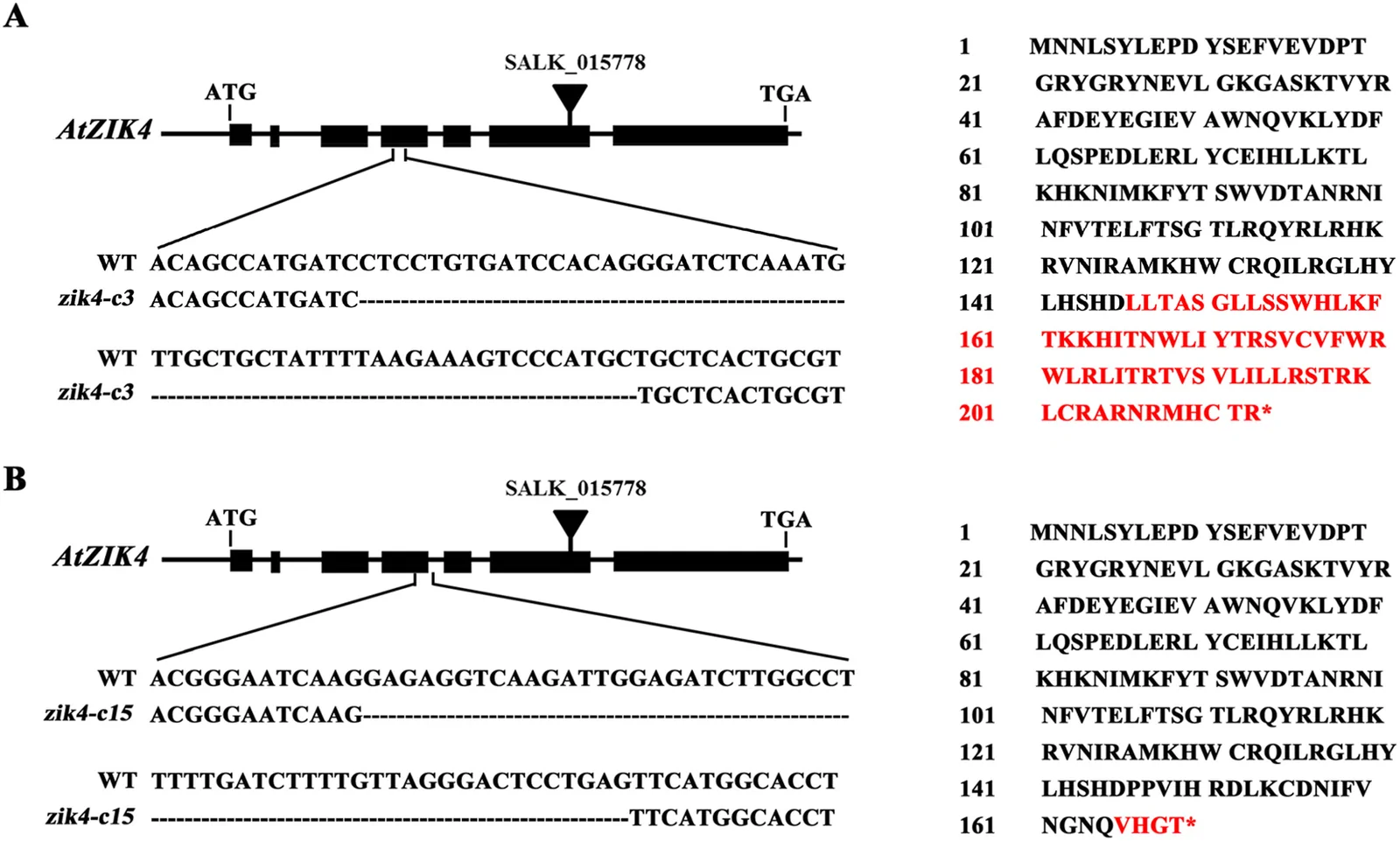
Generation and identification of *AtZIK4* knockout mutants via CRISPR/Cas9 technology. (A) Schematic diagram of the mutation site in the *zik4-c3* mutant obtained by CRISPR/Cas9-mediated knockout of *AtZIK4* in the Col-0 background, with the guideline indicating the deletion position and information in the mutant. The amino acid sequencing alignment result of the *zik4-c3* mutant is also shown. Red font indicates frameshift mutations caused by base deletions, and * represents premature termination. (B) Schematic diagram of the mutation site in the *zik4-c15* mutant obtained by CRISPR/Cas9-mediated knockout of *AtZIK4* in the Col-0 background, with the guideline indicating the deletion position and information in the mutant. The amino acid sequencing alignment result of the *zik4-c15* mutant is also shown. Red font indicates frameshift mutations caused by base deletions, and * represents premature termination.

Due to obtaining only one T-DNA insertion mutant of *AtZIK4*, a dual target vector was established using the CRISPR/Cas9 system to transform wild-type *A. thaliana* into knockout *AtZIK4*. Through PCR identification and sequencing analysis of the progeny, two knockout mutants, *zik4-c3* and *zik4-c1*5, with different deletion sites within the kinase domain were obtained. Both mutants exhibited large-fragment deletions, resulting in frameshift mutations and premature termination after the deletion sites. The knockout positions are shown in Figure 4.

**Figure 5:**
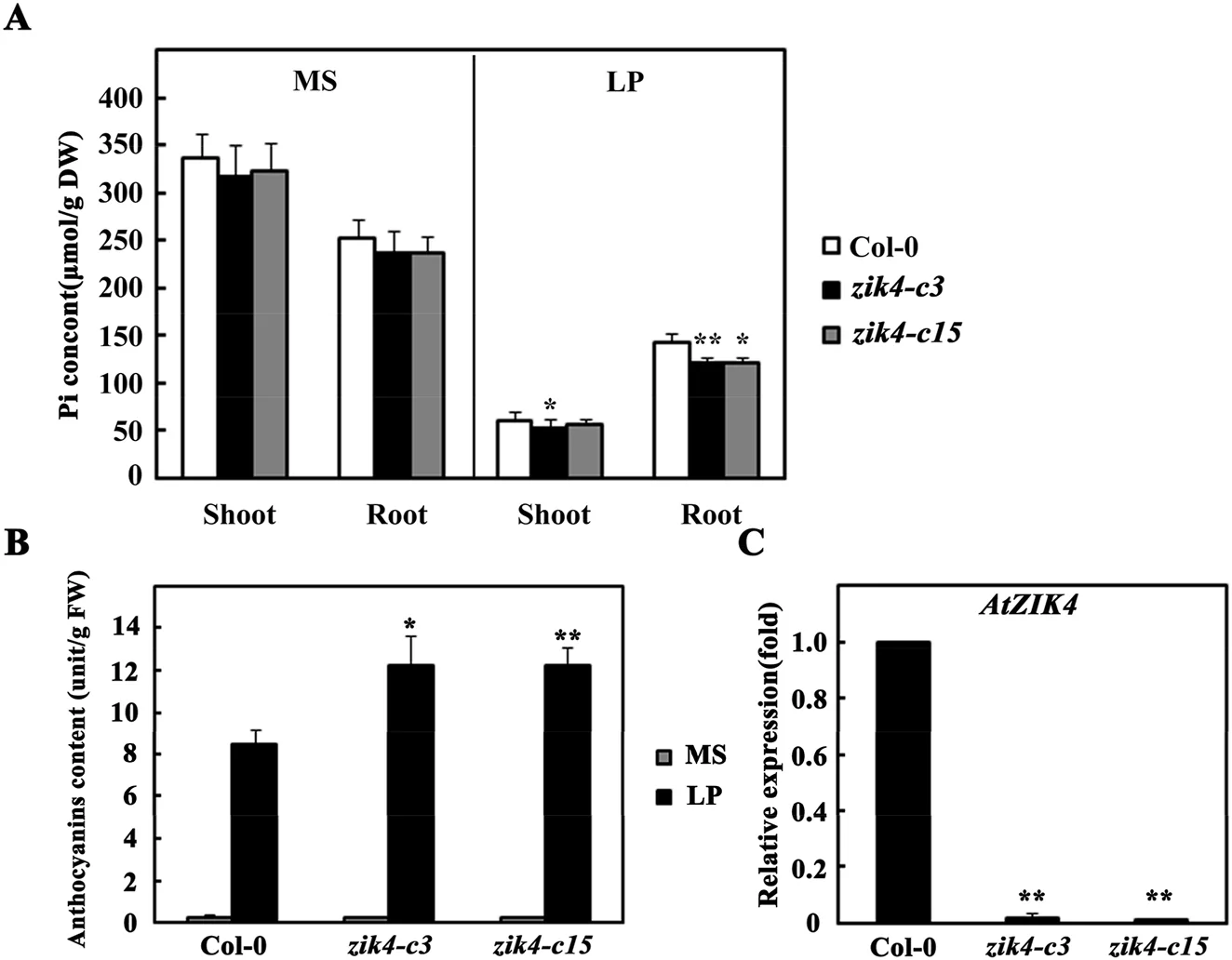
Attenuated tolerance to phosphate starvation in *AtZIK4* knockout mutants. Col-0, *zik4-c3* and *zik4-c15* seedlings vertically cultured on 1/2 MS medium for 7 days were transplanted to MS and LP media for further cultivation for 7 days. Root and shoot materials were collected for phosphate content determination, with results presented as mean values, error bars representing standard deviation, and three independent biological replicates (*t*-test was used to calculate significant differences, * indicates *P* < 0.05, ** indicates *P* < 0.01). (A) Phosphate content determination results in roots and shoots of Col-0, *zik4-c3* and *zik4-c15* seedlings. (B) Anthocyanin content determination results in Col-0, *zik4-c3* and *zik4-c15* seedlings. (C) Quantitative real-time PCR analysis of *AtZIK4* transcription levels in Col-0, *zik4-c3* and *zik4-c15* seedlings.

The expression of *AtZIK4* in *zik4-c3* and *zik4-c15* mutants was detected using real-time quantitative PCR. As shown in Figure 5(C), the transcriptional level of *AtZIK4* was significantly reduced in both mutants. The obtained *zik4-c3* and *zik4-c15* mutants could be used for subsequent phenotypic analysis. Seeds of Col-0, *zik4-c3* and *zik4-c15* were evenly sown on 1/2 MS medium, with about 1000 seeds per genotype. After 7 d of vertical light culture, the seedlings were transferred to MS and LP media for an additional 7 d of cultivation. Root and shoot materials were then collected for phosphorus content determination. As shown in Figure 5(A), phosphate content analysis revealed that seedlings grown in MS medium showed little difference in phosphate content. It had slightly reduced phosphate levels in both shoots and roots of *zik4-c3* and *zik4-c15* mutants. Under LP medium conditions, the phosphate content in shoots and roots of *zik4-c3* and *zik4-c15* mutants was lower than that in wild-type plants, with more significant differences in roots. In addition, the anthocyanin content was measured in crown tissues extracted from wild-type, *zik4-c3* and *zik4-c15* mutants after 7 d of continued cultivation on MS and LP media, respectively. As shown in Figure 5(B), the results demonstrated that *zik4-c3* and *zik4-c15* mutant seedlings grown on LP medium had significantly higher anthocyanin accumulation than the wild-type.

**Figure 6:**
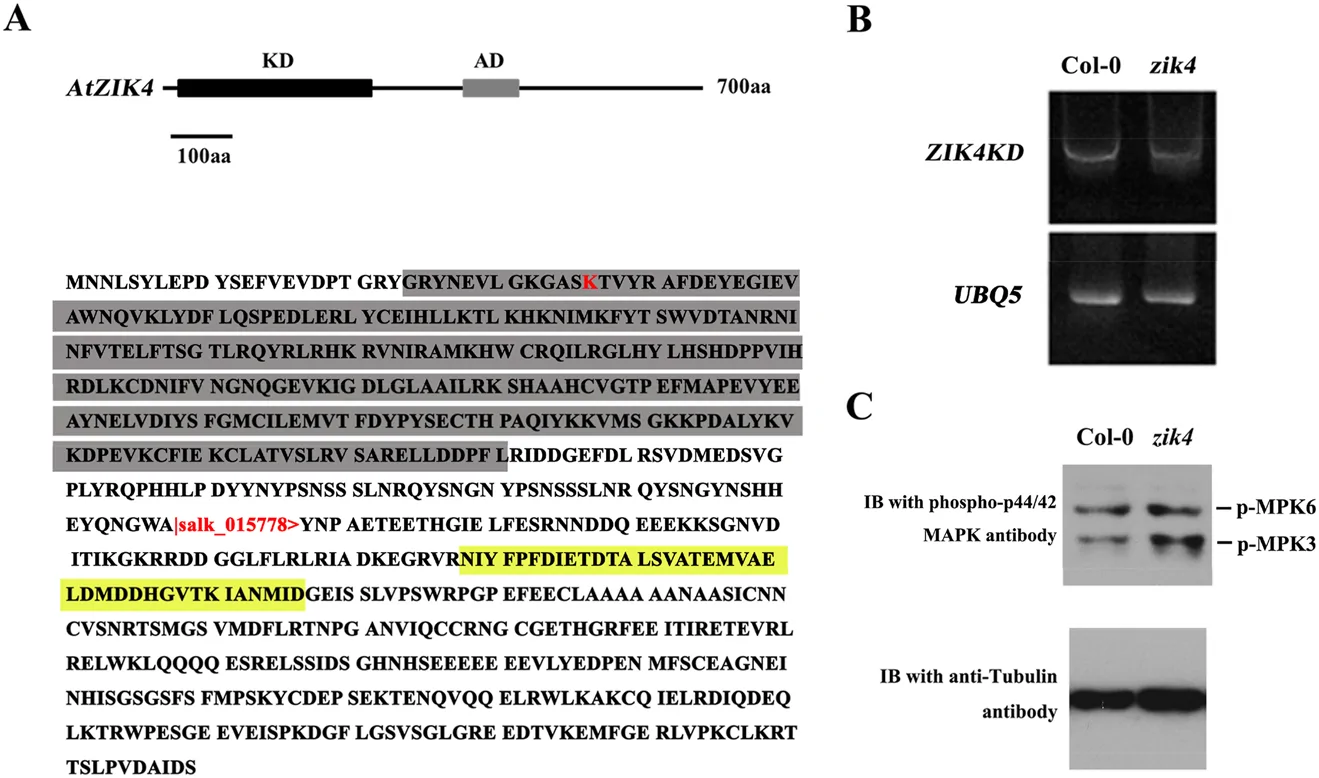
Sequence and activity analysis of *zik4* mutants. (A) Schematic diagram of AtZIK4 amino acid sequence structure and positions of T-DNA insertions in *zik4* mutants on the amino acid sequence. The upper row shows KD representing the kinase domain and AD representing the autoinhibitory domain. In the lower row amino acid sequence, gray background indicates the kinase domain, yellow background indicates the autoinhibitory domain, and red font-labeled SALK numbers mark T-DNA insertion sites. (B) RT-PCR analysis of kinase domain expression in *zik4* mutants. *UBQ5* is the internal control. (C) Detection of MPK3 and MPK6 activity in low-phosphate-treated seedlings of wild-type and mutant plants using anti-phospho-p44/42 MAPK antibody. Anti-Tubulin antibody is used as a protein loading control.

The above results, whether in phosphate content or anthocyanin determination, were opposite to those of the *zik4* T-DNA insertion mutant. The amino acid sequence of AtZIK4 and the sequencing results of the *zik4* mutant are analyzed. As shown in Figure 6(A), the knockout site of the CRISPR mutant is located in the kinase domain of AtZIK4, and the insertion position of the T-DNA mutant is located after the kinase domain and before the autoinhibitory region. The T-DNA insertion mutant kinase domain was identified using RT-PCR. As shown in Figure 6(B), the kinase domain of AtZIK4 was still expressed in the *zik4* mutant. Anti-Phospho-p44/42 MAPK antibody was used for immunoblot analysis to examine MAPK kinase activity in Arabidopsis materials under low phosphate treatment. The results in Figure 6(C) revealed that MPK3 and MPK6 activities is retained in the *zik4* mutant. It is speculated that the T-DNA insertion in *zik4* may affect the normal expression of the autoinhibitory region of AtZIK4, leading to a mutant with constitutive kinase activity. The above results indicate that *zik4* can accumulate more phosphate under LP conditions to adapt to low phosphate stress, and AtZIK4 may be involved in promoting phosphate accumulation.

### 3.3 AtZIK4 is induced by phosphate starvation stress

**Figure 7:**
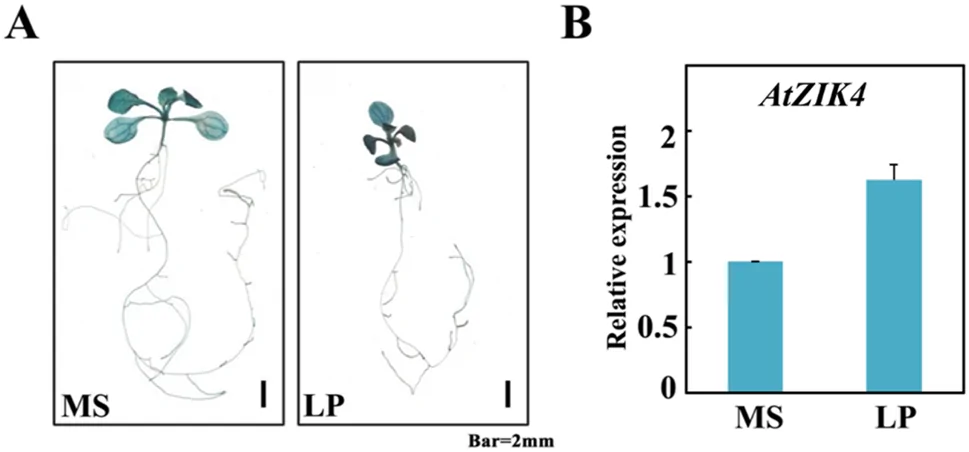
Analysis of the expression pattern of *AtZIK4* in seedlings. GUS staining was performed on *AtZIK4pro: GUS* transgenic seedlings to observe their tissue-specific expression patterns. Meanwhile, quantitative real-time PCR (Q-PCR) was used to detect the expression level of *AtZIK4* under different treatments. The results are presented as mean values, with error bars representing the standard deviation. (A) GUS staining results of transgenic seedlings which were vertically cultured on 1/2 MS medium for 7 d, then transplanted to MS and LP media respectively for another 7 d of vertical growth before sampling. Scale bar = 2 mm. (B) Wild-type seedlings grown in 1/2 MS liquid medium for 7 d were transferred to 1/2 MS (control), MS, and LP liquid media respectively for another 3 d of cultivation. Plant materials were harvested for RNA extraction to detect the transcription level of *AtZIK4*. The experiment was repeated three times, with the gene expression level of wild-type seedlings in 1/2 MS liquid medium as the baseline for calculating the fold change.

The expression pattern of a gene will affect its function. GUS histochemical staining was performed on the transgenic positive seedlings of the GUS reporter gene driven by *At-ZIK4* promoter sequence to observe the expression pattern of *AtZIK4* in the seedlings. At the same time, fluorescence quantitative PCR was used to detect the expression of *AtZIK4* in the seedlings. To observe the effect of phosphate starvation treatment on *AtZIK4* gene expression, *AtZIK4pro: GUS* seedlings cultured for 7 d were transferred to MS and LP medium respectively. The materials were taken for GUS staining after 7 d of vertical growth. The staining results are shown in Figure 7(A). The seedlings grown on LP medium showed deeper staining than those grown on MS medium, indicating that phosphate starvation stress induced the expression of *AtZIK4*. In addition, wild-type seedlings grown in 1/2MS medium for 7 d were transferred to 1/2MS medium, MS, and LP medium and continued to grow for 3 d. The materials were taken to identify the transcription level of *AtZIK4*. The transcription level of *AtZIK4* was induced in the seedlings (Figure 7(B)). The above results indicate that the overall expression of *AtZIK4* in seedlings is relatively high, and the transcription level of *AtZIK4* is induced by phosphate starvation stress.

### 3.4 AtZIK4 promotes the expression of Pi starvation response genes

Phosphate starvation stress can lead to changes in the transcription levels of various genes in plants. They include transcription factors from the WRKY, MYB, and bHLH families, high-affinity phosphate transporters, non-coding RNAs, and acid phosphatases. These genes are induced under phosphate starvation conditions to enhance the adaptability of plants to phosphate-deficient environments [9]. To investigate the role of AtZIK4 in the phosphate starvation response, real-time fluorescent quantitative PCR is used to measure the transcription levels of Pi starvation response genes in Col-0 and *zik4* mutant seedlings with phosphate starvation treatment. Given the numerous genes that respond to phosphate starvation stress, several representative genes are selected for analysis. PHT1; 1 and PHT1;4 are well-documented high-affinity phosphorus transporters of the PHT1 family that induced by phosphate starvation stress. They play a crucial role in phosphate uptake during such conditions [30]. WRKY75 encodes transcription factors belonging to the WRKY family, which are activated by phosphate starvation stress. The induced expression in the root system enhances phosphate uptake during phosphorus deficiency by affecting root structure [31]. MYB62 represents a transcription factor in the R2R3 MYB family and is expressed in leaves when subjected to phosphate starvation stress. This factor is localized in the nucleus, where it negatively regulates the response of *A. thaliana* to phosphate deficiency and help to maintain phosphate homeostasis [32]. PHO1 mainly promote the transport of phosphate from the outer epidermis to the xylem and is crucial for the movement of phosphate from the roots to aerial tissues [33]. The homologous genes *At4* and *IPS* are only 23 bases long and stimulated by phosphate starvation stress. They are partially complementary to miR399 that inhibits the silencing of the E2 ubiquitin-conjugating enzyme PHO2 by miR399 [34]. To investigate the metabolic response of mutants after phosphate starvation treatment, the expression of the anthocyanin synthase DFR encoding gene involved in anthocyanin biosynthesis was also analyzed.

**Figure 8:**
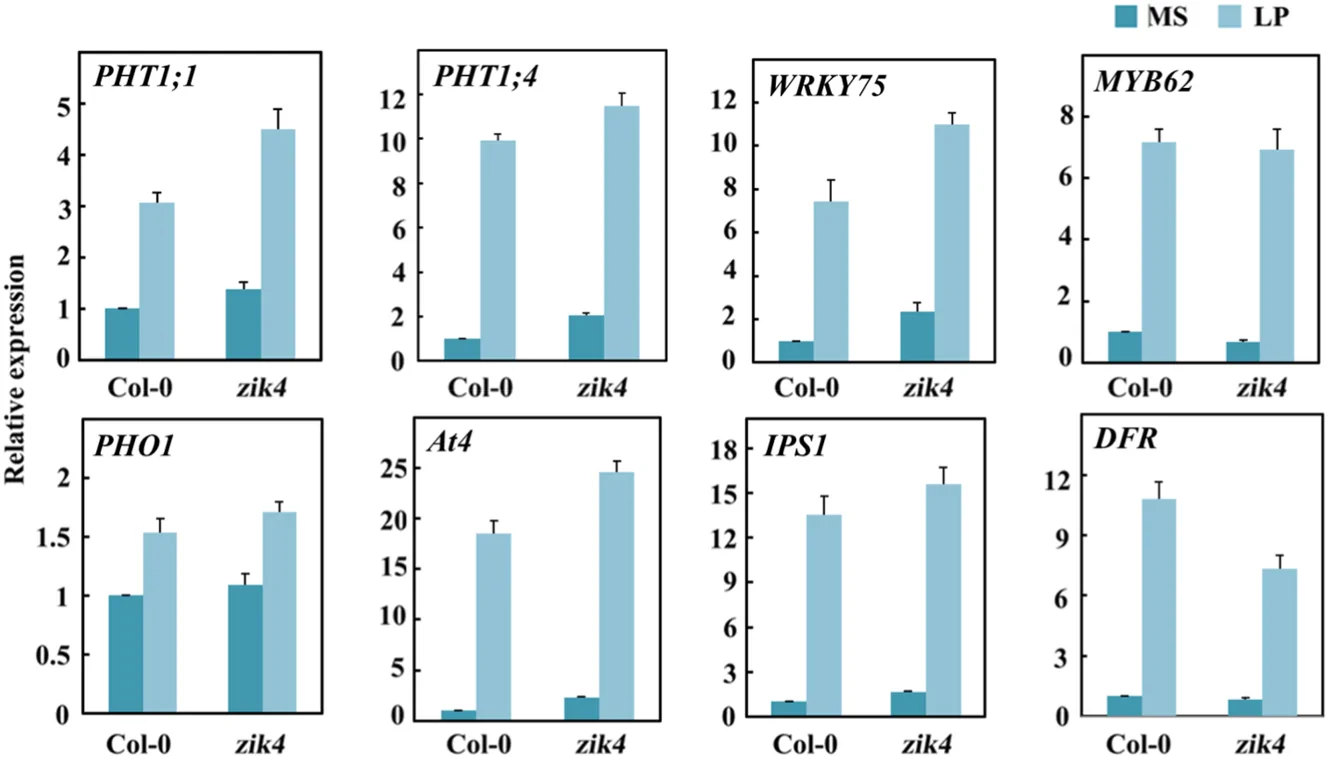
Transcription levels of phosphate starvation responsive genes in *zik4* mutants. Seedlings grown on 1/2 MS medium for 7 days were transplanted to MS and LP media respectively for another 7d cultivation. Each group comprised 15 independent biological samples. Total RNA was extracted from whole seedlings, and quantitative real-time PCR was performed to analyze the transcription levels of phosphate starvation responsive genes in each mutant. The results are presented as mean values with error bars indicating the standard deviation. The experiment was repeated three times, with the gene expression level of wild-type seedlings grown on MS medium as the baseline for calculating fold changes. The tissues were snap-frozen in liquid nitrogen and then transferred to an ultra-low temperature freezer at −80 °C for storage until RNA extraction. All samples were stored at −80 °C for no more than 3 months.

As shown in Figure 8, after wild-type seedlings were grown in LP medium for 7 d, the expression levels of phosphate starvation response genes were induced by low phosphate stress. The expression levels of phosphate uptake-related genes in *zik4* mutants such as *PHT1;1*, *PHT1;4*, and *WRKY75* were slightly increased on MS medium, and increased more significantly on phosphate starvation medium. Their levels induced by phosphate starvation stress were higher than that of wild type. The transcription level of MYB62, a transcription factor that inhibits the expression of PSI genes, was slightly lower than that of the wild type on MS medium. There is no significant change compared to the wild type on phosphate starvation medium. The levels of non-coding genes *At4* and *IPS1* induced by phosphate starvation were also significantly higher than those of the wild type. The expression of *PHO1* responsible for phosphate transport did not change significantly compared with the wild type. Compared with the wild type, the expression of the gene *DFR* encoding anthocyansin synthase in MS medium was slightly lower. Its induction level under phosphate starvation stress was significantly lower than that of the wild type. This indicates that AtZIK4 positively regulates the low-phosphate response of Arabidopsis seedlings by promoting the expression of phosphate uptake-related genes.

## 4 Conclusions

In this study, the function of ZIK4 in the Arabidopsis phosphate starvation response was investigated. Phenotypic analysis showed that the *zik4* mutant exhibited enhanced tolerance to Pi-deficient conditions. Since the kinase domain is still expressed in the T-DNA insertion mutant *zik4*, the CRISPR/Cas9 system was used to construct knockout mutants targeting two distinct sites of the AtZIK4 kinase domain. Observations of the LP phenotype revealed that the phosphate content in *zik4-c3* and *zik4-c15* was lower than that in the wild type. The insertion of T-DNA in *zik4* may affect the normal expression of the autoinhibitory region of AtZIK4, resulting in a mutant with constitutive kinase activity. Expression pattern analysis showed that *ZIK4* was induced under Pi starvation, and in the *zik4* mutant, several key phosphate uptake-related genes were significantly up-regulated under the same stress, as demonstrated by quantitative real-time PCR. These results indicate that ZIK4, a Pi starvation-induced MAPKKK, is a positive regulator of phosphate starvation stress. This study represents the preliminary identification of a MAPKKK within the ZIK family, which is associated with phosphate deficiency reactions.

The complete MAPK cascade is composed of tertiary protein kinases through step-by-step phosphorylation. Finding the complete MAPK cascade involved in the transmission of phosphate signals by protein kinase ZIK4 and solving how phosphate signals are transmitted to downstream response factors and regulatory elements will be an important task in the future.
